# Construction and validation of a cuproptosis-related lncRNA signature for the prediction of the prognosis of LUAD and LUSC

**DOI:** 10.1038/s41598-023-29719-1

**Published:** 2023-02-11

**Authors:** Yu Wang, Xu Xiao, Yan Li

**Affiliations:** 1 Department of Cardiology, Shenzhen Qianhai Taikang Hospital, Shenzhen, China; 2 Department of Esophageal surgery, Harbin Medical University Cancer Hospital, Harbin, China; 3 Department of Neurosurgery, Harbin Medical University Cancer Hospital, Harbin, China; 4 Department of Cardiology, Peking University Shenzhen Hospital, Shenzhen, China; 5 Department of Cardiology, Shenzhen Qianhai Taikang Hospital, No. 63 Qianwan Road 1, Shenzhen, 518054 Guangdong China

**Keywords:** Cancer genetics, Lung cancer, Computational biology and bioinformatics

## Abstract

Lung cancer is one of the most prevalent malignant tumors worldwide, with lung adenocarcinoma (LUAD) and lung squamous cell carcinoma (LUSC) accounting for the majority of cases. Cuproptosis, tumor immune microenvironment (TIME) and long non-coding RNA (lncRNA) have been demonstrated to be associated with tumorigenesis. The objective of the present study was to develop a novel cuproptosis-related lncRNA signature to assess the association between cuproptosis and TIME in patients with LUAD or LUSC and to predict prognosis. Based on the outputs of the least absolute shrinkage and selection operator regression model, a cuproptosis-related lncRNA signature was developed. Kaplan–Meier survival curves were generated to confirm the predictive ability of the signature. Univariate and multivariate analysis was also performed to determine the association between overall survival and this signature and other clinical characteristics, and a nomogram was created. Additionally, the relationship between the signature, TIME, tumor mutation burden and m6A methylation was established. The results of the present study revealed that 8 cuproptosis-related lncRNAs were associated with the prognosis of patients with LUAD and LUSC. This novel cuproptosis-related lncRNA signature is associated with TIME and m6A methylation in LUAD and LUSC and can predict prognosis with accuracy.

## Introduction

Lung cancer has become the most common malignant tumor worldwide and is associated with both high morbidity and mortality according to the GLOBOCAN 2018 statistics^1^. Furthermore, non-small cell lung cancer (NSCLC) accounts for > 85% of lung cancer cases^2^. The most common histologic subtypes of NSCLC are lung squamous cell carcinoma (LUSC) and lung adenocarcinoma (LUAD)^3^. Additionally, as lung cancer has complex molecular mechanisms, targeted therapies may be ineffective for some patients, posing a significant challenge for lung cancer treatment^4^. Therefore, identifying novel biomarkers for LUAD or LUSC is critical to help develop therapeutic decisions and guide individualized prognostics.

Copper homeostasis is crucial for numerous physiological processes as an essential cofactor. Tsvetkov et al. discovered a novel mode of cell death known as ‘copper-dependent cell death’ (termed cuproptosis). Cuproptosis is characterized as a copper-triggered modality of mitochondrial cell death and is a type of nonapoptotic cell death pathway. The pathogenic mechanism of cuproptosis is that direct copper binding to lipidated tricarboxylic acid (TCA) cycle components results in the loss of iron-sulfur cluster proteins and lipid acylated protein aggregation, which in turn causes proteotoxic stress and cell death^5^. Cuproptosis may contribute to the modulation of several illnesses, including cancer. However, it is still unclear if cuproptosis is associated with LUAD or LUSC.

Long non-coding RNAs (lncRNAs), which are defined as transcripts with a length > 200 nucleotides and little to no ability to code for proteins, have a variety of functions in controlling the expression levels of proteins in different types of human malignancies^6^. Enhanced lncRNA function or expression may serve a significant role in a variety of cancer-related diseases^7^. lncRNA influences numerous biological processes through a variety of mechanisms, including cell proliferation, cell differentiation, metastatic progression, proliferation or apoptosis, sex-chromosome dosage compensation, the maintenance of genome stability, and the stemness and modulation of metabolism^8–10^, particularly in cancer. Emerging evidence suggests that cuproptosis may be regulated by several lncRNAs. According to the research of Liu et al., LINC01232 upregulates RAB22A through interaction with microRNA-204-5p to induce clear cell renal cell carcinoma^11^. Cuproptosis-associated lncRNAs may be associated with head and neck squamous cell carcinoma^12^, cutaneous melanoma^13^, oral squamous cell carcinoma^14^ and other cancer types^15,16^. There are some researches shows lncRNAs can influent cuproptosis in LUAD^17,18^. However, at present, it is unclear how cuproptosis-associated lncRNAs affect tumor immunity (TIME) and the prognosis of LUAD and LUSC at the same time.

In the present study, a novel predictive model for patients with LUAD and LUSC was developed based on cuproptosis-related lncRNAs. Patients with LUAD and LUSC may benefit from the ability of the model to forecast the long-term prognosis and provide tailored treatment methods.

## Materials and methods

### Expression data and clinical information of patients

The Cancer Genome Atlas (TCGA) database (https://portal.gdc.cancer.gov/) was used to gather the clinical features and transcriptome information of the LUAD and LUSC samples. A total of 1041 lung cancer tissues and 108 normal tissues were included in the samples of transcriptome data. A total of 1002 patients with LUAD or LUSC provided the clinical data. Perl (https://www.perl.org, version 5.32.1) was used to collate the clinical details.

### Differentially expressed cuproptosis-related lncRNAs

**Figure 1:**
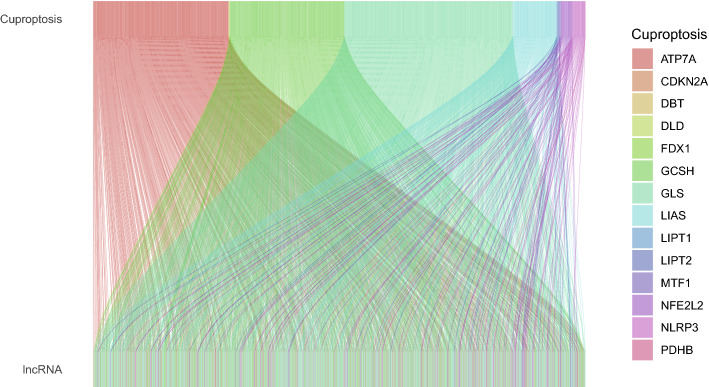
Sankey diagram showing the association between 1138 cuproptosis-related lncRNAs and 19 cuproptosis genes. lncRNA, long non-coding RNA.

A total of 19 genes were selected to be cuproptosis-related genes (NFE2L2, NLRP3, ATP7B, ATP7A, SLC31A1, FDX1, LIAS, LIPT1, LIPT2, DLD, DLAT, PDHA1, PDHB, MTF1, GLS, CDKN2A, DBT, GCSH and DLST). A total of 16,773 lncRNAs were determined using the transcriptome data of 1149 LUAD or LUSC samples. The cuproptosis-related lncRNAs were extracted using the R package ‘limma’ according to the threshold (coefficient > 0.4 and P < 0.001). This progress was visualized as a Sankey diagram (Fig. 1). The details of the association between cuproptosis-related lncRNAs and cuproptosis genes are shown in Table S1. The Sankey diagram was created using the R packages ‘dplyr’, ‘ggalluvial’ and ‘ggplot2’.

### Construction and assessment of the accuracy of the cuproptosis-related lncRNA signature

**Figure 2:**
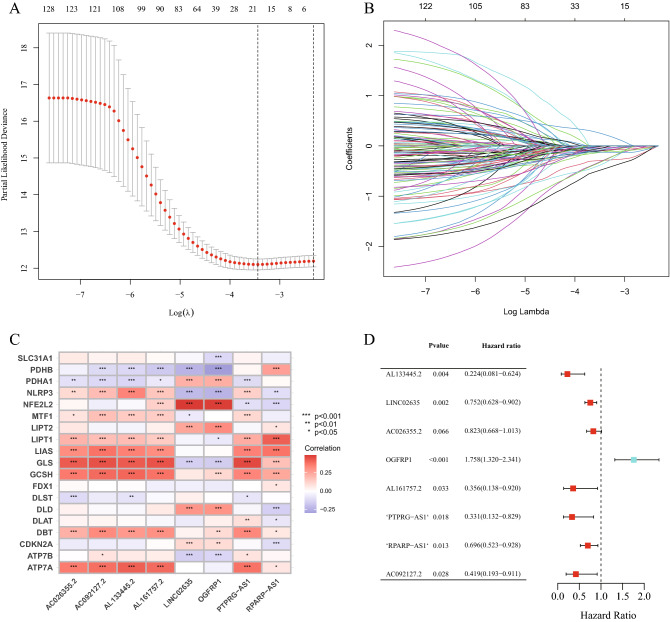
Construction of the cuproptosis-related lncRNA signature. (**A**) Confidence intervals are displayed for each lambda. (**B**) Partial likelihood deviation for various variable counts. The independent variable's coefficient is represented by the vertical axis, while the independent variable's log value is represented by the horizontal axis. (**C**) Correlations between signature lncRNAs and cuproptosis-related genes. (**D**) Multivariate Cox regression analysis of 8 signature lncRNAs. lncRNA, long non-coding RNA.

The 501 training samples and 501 validation samples from the 1002 tumor samples acquired from TCGA were randomly divided into two groups. Table 1 shows all sample information. The risk signature was created using the Training Set. The validity of the prognosis was tested using the Validation Set and the Entire Set. From the entire set of cuproptosis-related lncRNAs, 133 were selected for univariate Cox proportional hazard regression analysis and were found to be significantly associated with the overall survival (OS) of patients with lung cancer (P < 0.05; Table S2). A total of 8 lncRNAs were employed to create the prognostic signature of patients with lung cancer, and the 133 cuproptosis-related prognostic lncRNAs were subjected to a least absolute shrinkage and selection operator (LASSO) test and multivariate analysis to reduce error rates (Fig. 2). The risk score was calculated as follows: [Expression level of Gene 1] + [Expression level of Gene 2] + [Expression level of Gene n] + [Expression level of Gene n] coefficient. The aforementioned algorithm was used to determine the risk scores of the patients. The median of the risk scores was used to divide the patients into high- and low-risk groups. The Kaplan–Meier survival curves, c-index, principal component analysis (PCA) and receiver operating characteristic (ROC) curves were used to compare the patient survival rates in the low- and high-risk groups using the ‘survival’, ‘survminer’, ‘rms’, ‘pec’ and ‘timeROC’ R packages to confirm the accuracy of this signature.

**Table 1:** Clinical information of the entire set, training set and validation set.

Variables	Group	Entire set (n = 1002)	Training set (n = 501)	Validation set (n = 501)	P-value
Age	≤ 65	428 (42.71%)	220 (43.91%)	208 (41.52%)	0.5896
	> 65	558 (55.69%)	276 (55.09%)	282 (56.29%)	
	Unknow	16 (1.6%)	5 (1%)	11 (2.2%)	
Gender	Female	401 (40.02%)	195 (38.92%)	206 (41.12%)	0.5191
	Male	601 (59.98%)	306 (61.08%)	295 (58.88%)	
Stage	Stage I	514 (51.3%)	262 (52.3%)	252 (50.3%)	0.6556
	Stage II	279 (27.84%)	135 (26.95%)	144 (28.74%)	
	Stage III	164 (16.37%)	86 (17.17%)	78 (15.57%)	
	Stage IV	33 (3.29%)	14 (2.79%)	19 (3.79%)	
	Unknow	12 (1.2%)	4 (0.8%)	8 (1.6%)	
T stage	T1	283 (28.24%)	154 (30.74%)	129 (25.75%)	0.0919
	T2	559 (55.79%)	262 (52.3%)	297 (59.28%)	
	T3	115 (11.48%)	65 (12.97%)	50 (9.98%)	
	T4	42 (4.19%)	20 (3.99%)	22 (4.39%)	
	Unknow	3 (0.3%)	0 (0%)	3 (0.6%)	
M stage	M0	745 (74.35%)	369 (73.65%)	376 (75.05%)	0.6456
	M1	32 (3.19%)	14 (2.79%)	18 (3.59%)	
	Unknow	225 (22.46%)	118 (23.55%)	107 (21.36%)	
N stage	N0	643 (64.17%)	337 (67.27%)	306 (61.08%)	0.1652
	N1	223 (22.26%)	102 (20.36%)	121 (24.15%)	
	N2	111 (11.08%)	51 (10.18%)	60 (11.98%)	
	N3	7 (0.7%)	5 (1%)	2 (0.4%)	
	Unknow	18 (1.8%)	6 (1.2%)	12 (2.4%)	

*TCGA* The Cancer Genome Atlas.

### Construction of the nomogram

The nomogram and calibration curve were constructed using the R packages ‘survival’, ‘regplot’ and ‘rms’. The nomogram predicted the 1-year, 3-year and 5-year survival rates based on the risk score and other clinical factors.

### Analyses of functional enrichment

Using the R packages ‘clusterProfiler’, ‘org.Hs.eg.db’, ‘enrichplot’, ‘ggplot2’, ‘RColorBrewer’, ‘dplyr’, ‘ggpubr’ and ‘ComplexHeatmap’, two types of functional enrichment analyses, Gene Ontology (GO; http://www.geneontology.org/) and Kyoto Encyclopedia of Genes and Genomes (KEGG; http://www.genome.jp/kegg/) analyses, were performed. Based on the threshold (P < 0.05), GO terms and KEGG signaling pathways were determined to be enriched.

### Analysis of tumor mutation burden (TMB)

TMB data were saved in the mutation annotation format and retrieved from TCGA (https://portal.gdc.cancer.gov/). The R packages ‘TCGAmutations’ and ‘maftools’ were used to perform TMB analysis.

### Analysis of TIME

The immune infiltration of cells was calculated using TIMER, CIBERSORT, CIBERSORT-ABS, QUANTISEQ, MCP-counter, XCELL and EPIC algorithms. The immune function analysis and immune checkpoints were calculated using the R packages ‘GSEABase’ and ‘limma’, respectively.

### Analysis of m6A methylation

The 12 m6A methylation-related genes were selected based on previous studies^19,20^. The R packages ‘limma’, ‘reshape2’, ‘ggplot2’ and ‘ggpubr’ were applied to compare the expression levels of 12 genes and draw the plot.

### Statistical analysis

The R package ‘survival’ was used to perform univariate and multivariate Cox regression calculations in order to validate the prediction of the signature and clinicopathological variables. Differences between clinical characteristics were identified using an independent t-test by SPSS 22.0 statistical software (IBM Corp.). P < 0.05 was considered to indicate a statistically significant difference. All of R packages used in current article were performed by R software (https://www.r-project.org/, R version 4.2.1).

## Results

### Construction of the cuproptosis-related lncRNA signature

A total of 19 genes were selected to be cuproptosis genes based on earlier research^5,16^. The transcriptome data were downloaded for the LUAD and LUSC cohorts in TCGA and were divided into mRNAs and lncRNAs. Subsequently, a correlation test was performed to extract cuproptosis-related lncRNAs based on the threshold (coefficient > 0.4 and P < 0.001). Finally, a total of 1138 lncRNAs were associated with cuproptosis and were determined to be cuproptosis-related lncRNAs (Fig. 1, Table S1).

The clinical data of 1002 patients with lung cancer were downloaded from TCGA as an entire set to identify the cuproptosis-related lncRNAs that were associated with patient prognosis. The clinical characteristics of these 1002 samples are shown in Table 1. Subsequently, the entire set (n = 1002) was randomly divided into a training set (n = 501) and a validation set (n = 501). Univariate analysis was performed on the training set, which determined that 133 cuproptosis-related lncRNAs were significantly associated with the prognosis of patients with lung cancer (P < 0.05, Fig. S1, Table S2). To minimize the error rate, LASSO analysis and multivariate analysis were performed. Finally, 8 cuproptosis-related prognostic lncRNAs were extracted (Fig. 2A,B). In multivariate Cox regression analysis, the risk score was created by linearly combining the expression levels of the 8 cuproptosis-related prognostic lncRNAs, weighted by their relative coefficient (Fig. 2D, Table 2) as follows: Risk score = (− 1.495239459 * AL133445.2) + (− 0.284516815 * LINC02635) + (− 0.195045442 * AC026355.2) + (0.563925195 * OGFRP1) + (− 1.031936125 * AL161757.2) + (− 1.105927746 * PTPRG-AS1) + (− 0.361962738 * RPARP-AS1) + (− 0.868805559 * AC092127.2). Figure 2C shows the relationships between the 19 cuproptosis genes and 8 cuproptosis-related prognostic lncRNAs.

**Table 2:** Coefficients and multivariable Cox model results of 8 lncRNAs in the cuproptosis-related prognostic signature.

Id	Coef	HR	HR.95L	HR.95H	P-value
AL133445.2	− 1.495239459	0.224194913	0.080584712	0.623733182	0.004181331
LINC02635	− 0.284516815	0.752377704	0.627776666	0.901709542	0.002070143
AC026355.2	− 0.195045442	0.822797268	0.668100262	1.013313993	0.066431958
OGFRP1	0.563925195	1.757557735	1.319616711	2.340838188	0.00011492
AL161757.2	− 1.031936125	0.356316419	0.137966465	0.920233699	0.03303303
PTPRG-AS1	− 1.105927746	0.330903745	0.132078602	0.829031253	0.018270484
RPARP-AS1	− 0.361962738	0.696308313	0.522736813	0.927513148	0.013347615
AC092127.2	− 0.868805559	0.419452261	0.193185792	0.910730531	0.028066597

### Cuproptosis-related lncRNA signature and the survival of patients with LUAD and LUSC

**Figure 3:**
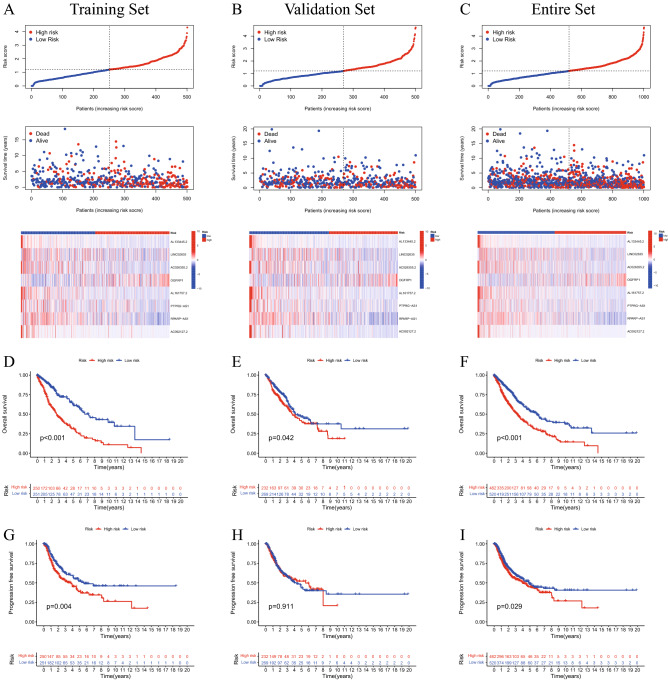
Prognosis of patients with lung cancer is accurately predicted by a cuproptosis-related lncRNA signature. The 8 cuproptosis-related lncRNAs were expressed in low- and high-risk groups in the training set (**A**), validation set (**B**) and entire set (**C**), with respect to risk score distribution, survival status and expression. K-M survival curve analyses of OS of patients with lung cancer in the training set (**D**), validation set (**E**) and entire set (**F**). K-M survival curve analyses of PFS of patients with lung cancer in the training set (**G**), validation set (**H**) and entire set (**I**). K-M, Kaplan–Meier; lncRNA, long non-coding RNA; OS, overall survival; PFS, progression-free survival.

According to the median risk score, patients in the training set, validation set and full set were divided into high- and low-risk groups (Fig. 3A–C). The findings of the present study demonstrated that patients in the low-risk group had a longer OS time than those in the high-risk group (p < 0.05; Fig. 3D–F). Additionally, the low-risk patients in the training set and entire set exhibited longer PFS than those patients with high risk scores (P < 0.05, Fig. 3G,I). However, the patients in the validation set did not show the same trend (P > 0.05, Fig. 3H).

### Cuproptosis-related lncRNA signature as an independent prognostic factor in patients with LUAD and LUSC

**Figure 4:**
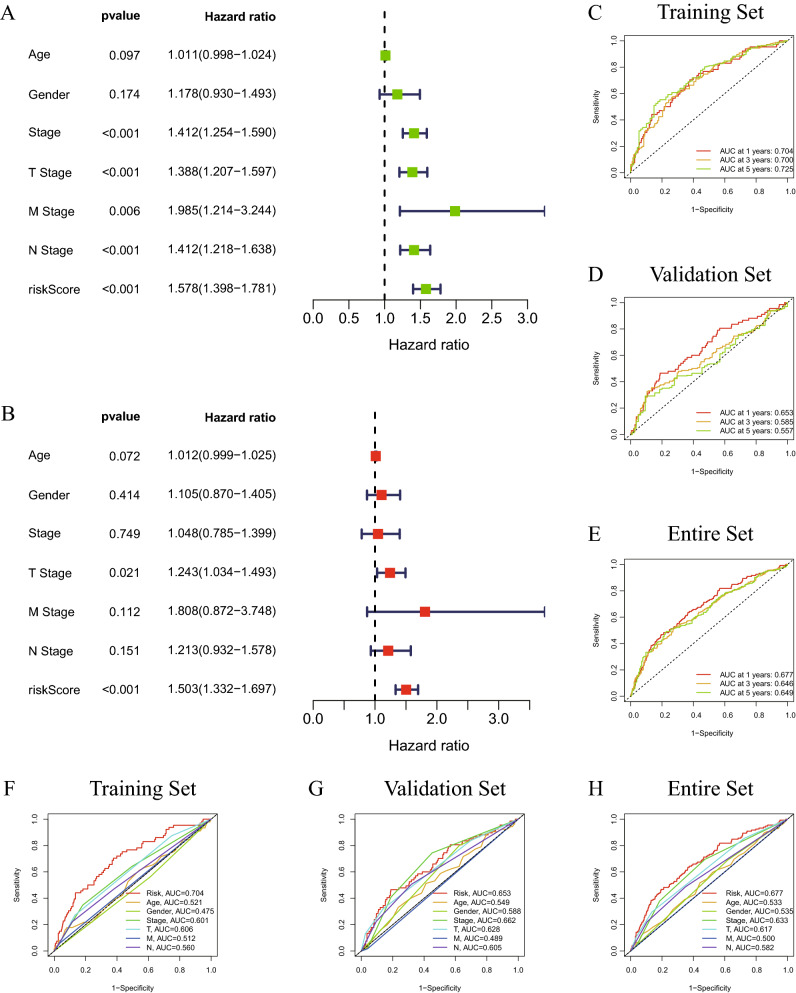
Forest diagrams of univariate (**A**) and multivariate (**B**) Cox regression analyses illustrate that the risk score is an independent prognostic factor in lung cancer. The ROC curves of the 1-year, 2-year and 5-year survival rate in the training set (**C**), validation set (**D**) and entire set (**E**). The ROC curves of the risk score, age, sex, stage, T stage, N stage and M stage in the training set (**F**), validation set (**G**) and entire set (**H**). ROC, receiver operating characteristic.

**Figure 5:**
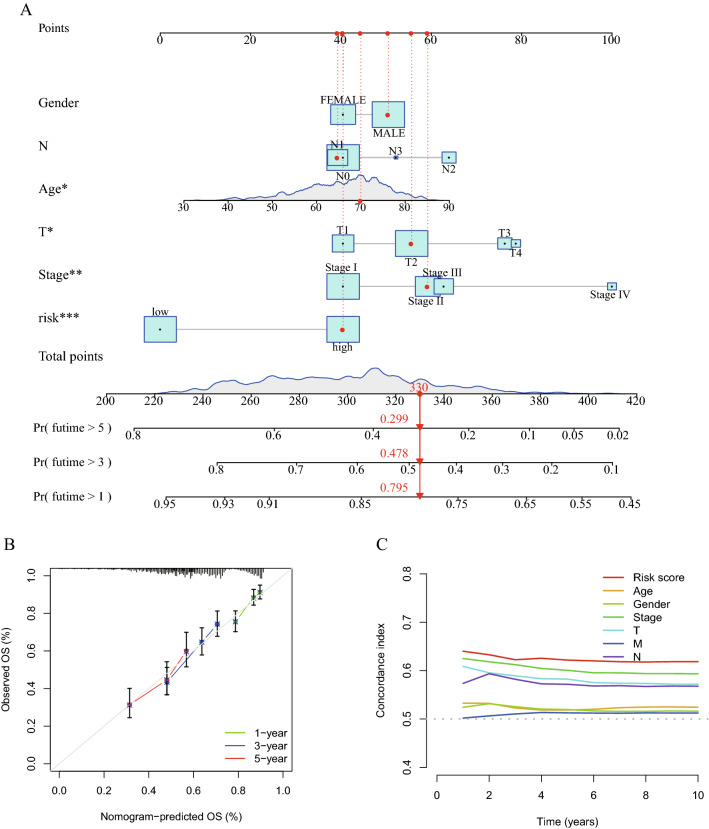
(**A**) A nomogram to predict 1-, 3- and 5-year OS of patients with lung cancer. (**B**) Nomogram-based calibration curves that show consistency between predicted and detected 1-, 3- and 5-year survival. (**C**) The c-index curve to compare the predictive ability of the risk score and the other clinical factors. OS, overall survival.

To determine if the cuproptosis-related lncRNA signature may serve as an independent predictor of the survival of patients with lung cancer, univariate (Fig. 4A) and multivariate (Fig. 4B) analyses of the risk score, OS and other clinical indicators were performed (Table 3). The results of multivariate analysis performed on the entire set suggested that the risk score and T stage may act independently as prognostic indicators for patients with lung cancer (P < 0.05). Furthermore, the accuracy of the cuproptosis-related lncRNA signature was examined using time-dependent ROC curves (Fig. 4C). The under regions of the training set for 1-year, 3-year and 5-year survival were 0.704, 0.700 and 0.725, respectively, demonstrating the potential predictive value of the cuproptosis-related lncRNA signature for patients with lung cancer. Additionally, using the data from the validation set (Fig. 4D) and the complete set, similar ROC curves were created (Fig. 4E). The present study also compared the predictive ability of the risk score with the other clinical characteristics in the training set (Fig. 4F), validation set (Fig. 4G) and entire set (Fig. 4H) using ROC curves. The under areas of the risk score were larger than those of the other clinical factors in the different sets. The c-index of the risk score was greater than that of the other clinical factors (Fig. 5C). In order to produce a precise tool to assess the survival of patients with lung cancer, a nomogram was also created in line with the risk score and the aforementioned clinicopathological indicators (Fig. 5A). In particular, the calibration curves of the prognostic nomogram indicated that the predicted and actual survival rates at 1, 3 and 5 years were generally consistent (Fig. 5B). The cuproptosis-related lncRNA signature had a high level of predictive power according to all results.

**Table 3:** Univariate and multivariate analyses with Cox proportional hazard model.

Characteristics	Univariate analysis				Multivariate analysis			
	HR	HR.95L	HR.95H	P-value	HR	HR.95L	HR.95H	P-value
Age	1.010813793	0.998054561	1.02373614	0.097013304	1.011886345	0.998953982	1.024986128	0.071782626
Gender	1.178261447	0.930052697	1.492711157	0.174098951	1.105180074	0.869617581	1.404551866	0.413524318
Stage	1.412311364	1.254116096	1.590461518	1.23E-08	1.048298253	0.785299793	1.39937542	0.748933339
T stage	1.388384222	1.207014884	1.59700661	4.34E-06	1.242539912	1.033986625	1.493158032	0.020531819
M stage	1.984910983	1.214435109	3.244201011	0.006237697	1.807612299	0.871839952	3.747777578	0.111540311
N stage	1.412365976	1.218044459	1.637688703	4.84E-06	1.212805299	0.931835268	1.578494337	0.151314554
Risk score	1.578316247	1.398413772	1.78136273	1.46E-13	1.503379388	1.331555912	1.697374901	4.57E-11

*HR* hazard ratio.

### Predictive ability of the cuproptosis-related lncRNA signature in different clinical subgroups

**Figure 6:**
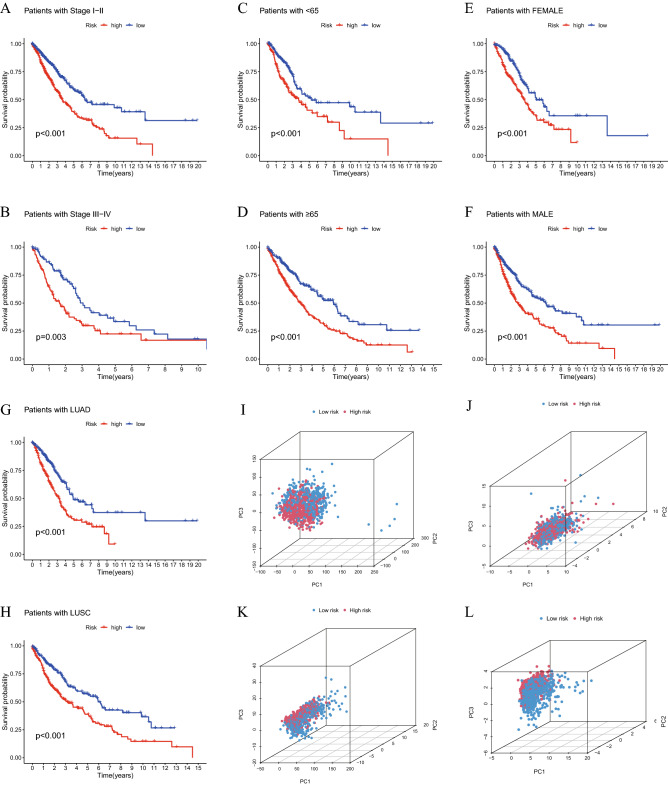
K-M survival curves of patients. Stage I-II (**A**), stage III-IV (**B**), age < 65 years (**C**), age ≥ 65 years (**D**), female (**E**) and male (**F**), LUAD (**G**) and LUSC (**H**). PCA diagrams according to the expression of all genes (**I**), cuproptosis-related genes (**J**), cuproptosis-related lncRNAs (**K**) and 8 signature lncRNAs (**L**). K-M, Kaplan–Meier; lncRNA, long non-coding RNA; PCA, principal component analysis.

To explore if the predictive ability of the cuproptosis-related lncRNA signature could be affected by clinical factors, three common clinical characteristics (stage, age and sex) were selected as criteria to divide patients into different subgroups (stage I-II and III-IV; age < 65 and ≥ 65 years; female and male; LUAD and LUSC). The findings showed that all subgroups of high-risk patients had worse OS (P < 0.05; Fig. 6A–H), which illustrated that the predictive ability of the present signature was stable in all these subgroups. PCA was used to detect the grouping ability of the cuproptosis-related lncRNA signature. According to all genes (Fig. 6I), cuproptosis-related genes (Fig. 6J) and cuproptosis-related lncRNAs (Fig. 6K), no significant differences were found. However, the high- and low-risk patients could be successfully differentiated by 8 cuproptosis-related lncRNAs (Fig. 6L). Therefore, the cuproptosis-related lncRNA signature could classify high-risk patients with lung cancer in different clinical factor subgroups.

### Cuproptosis-related lncRNA signature and immune cell infiltration

**Figure 7:**
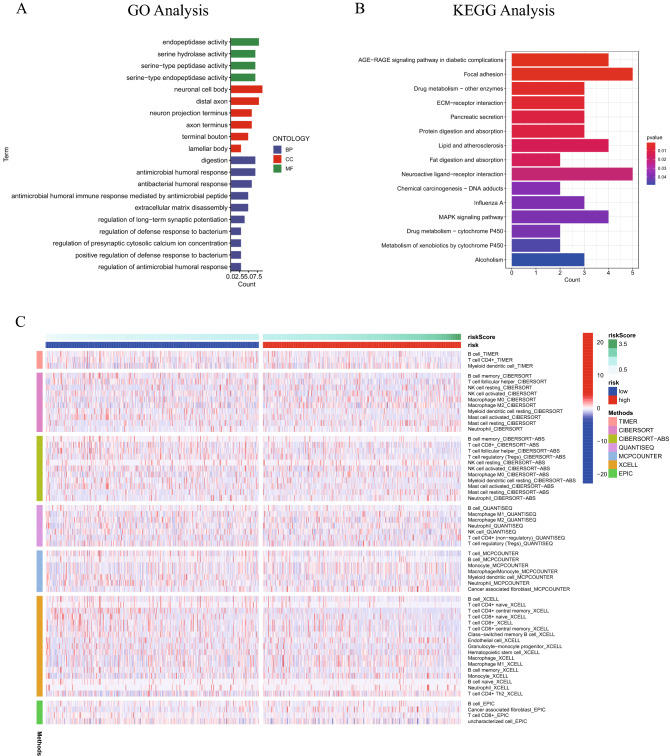
GO enrichment analysis (**A**) and KEGG enrichment analysis (**B**) of differently expressed genes between the high- and low- risk patients. (**C**) Heatmap of differently infiltrated immune cells between high- and low-risk patients according to the TIMER, CIBERSORT, CIBERSORT-ABS, QUANTISEQ, MCP-counter, XCELL and EPIC algorithms. GO, Gene Ontology; KEGG, Kyoto Encyclopedia of Genes and Genomes.

In terms of the mechanism via which the cuproptosis-related lncRNA signature was associated with the prognosis of patients with lung cancer, 155 genes were differentially expressed in high- and low-risk samples according to the criteria (log fold change > 1, FDR < 0.05, Table S3). Two types of functional enrichment analyses, GO analysis and KEGG analysis, were performed. The GO analysis illustrated that most differently expressed genes were enriched in ‘antimicrobial humoral response’, ‘antibacterial humoral response’ and ‘antimicrobial humoral immune response mediated by antimicrobial peptide’ (Fig. 7A). The KEGG analysis^21–23^ demonstrated that ‘AGE-RAGE signaling pathway’, ‘Focal adhesion’ and ‘Drug metabolism—other enzymes’ were enriched by differently expressed genes (Fig. 7B). TIME, TIMER, CIBERSORT, CIBERSORT-ABS, QUANTISEQ, MCP-counter, XCELL and EPIC were used to calculate the relative proportion of immune cell infiltration in lung cancer to ascertain the roles of the aforementioned signature. The differently infiltrated immune cell types between the two groups are shown in Fig. 7C and Table S4 (P < 0.05).

### Cuproptosis-related lncRNA signature and immune cell functions and checkpoints

**Figure 8:**
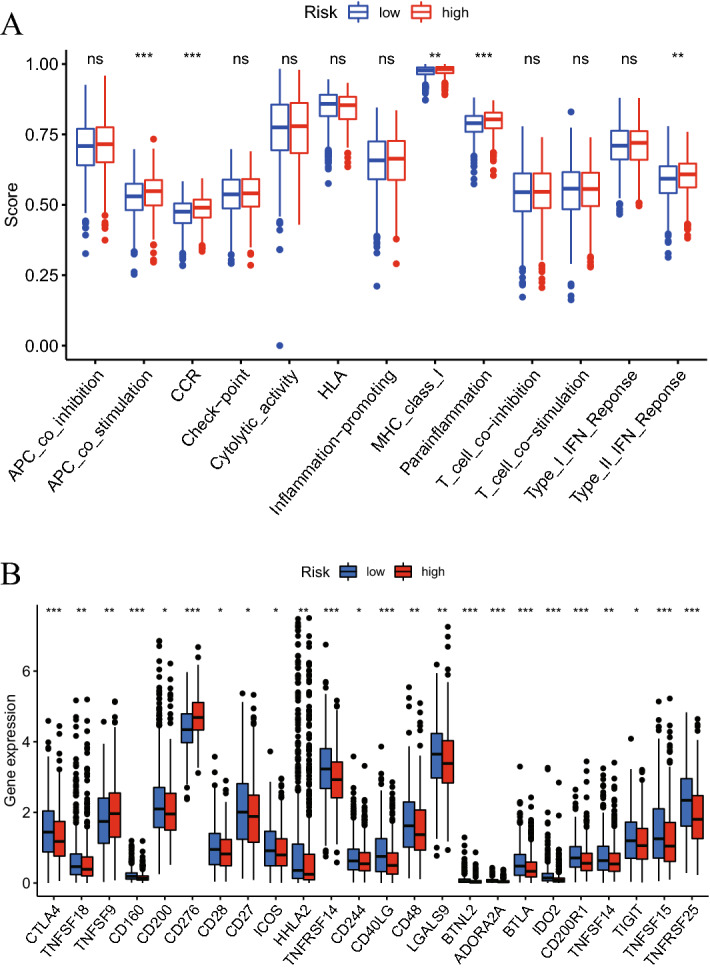
(**A**) ssGSEA scores of 13 immune-related functions in high- and low-risk patients. (**B**) Expression levels of 24 immune checkpoints in high- and low-risk patients. *P < 0.05, **P < 0.01, ***P < 0.001.

To further explore the association between the cuproptosis-related lncRNA signature and the immune system, single-sample Gene Set Enrichment Analysis (ssGSEA) was used to investigate the differences in immune function between high- and low-risk patients. The results of ssGSEA demonstrated significant differences in ‘APC_co_stimulation’, ‘cytokine receptor (CCR)’, ‘MHC_class_I’, ‘Parainflammation’ and ‘Type_II_IFN_Reponse’ (Fig. 8A). Additionally, 24 types of immune checkpoint expression levels were compared between high- and low-risk patients (Fig. 8B). The results illustrated that all 24 immune checkpoints were expressed differently in high- and low-risk patients, which demonstrated that the cuproptosis-related lncRNA signature had the ability to predict immune checkpoint responses.

### Cuproptosis-related lncRNA signature and TMB

**Figure 9:**
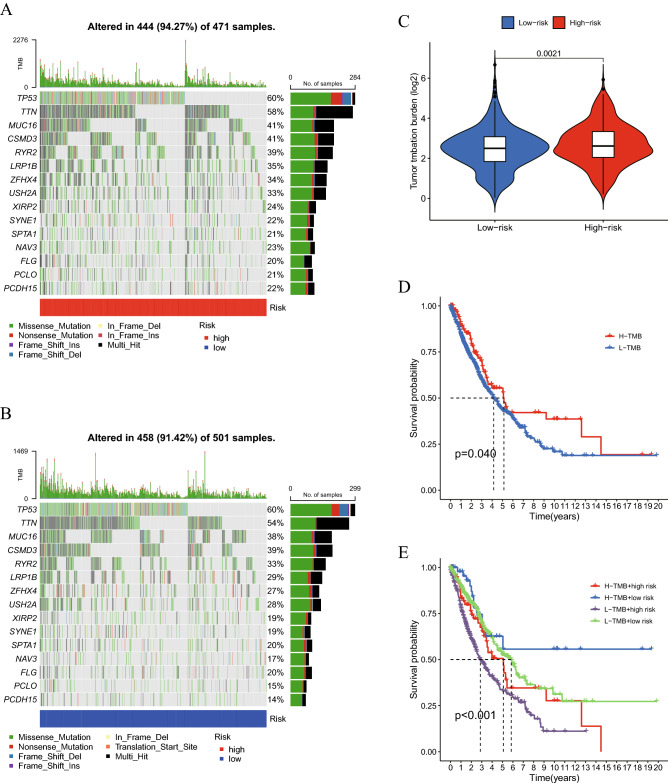
Mutation profile in patients with (**A**) high risk and (**B**) low risk. (**C**) Relationship between TMB and the cuproptosis-related lncRNA signature. (**D**) K-M curves in the high-TMB and low-TMB groups. (**E**) K-M curves in the high-TMB + high-risk, high-TMB + low-risk, low-TMB + high-risk and low-TMB + low-risk groups. K-M, Kaplan–Meier; lncRNA, long non-coding RNA; TMB, tumor mutation burden; OS, overall survival.

TMB serves as a biomarker for prognosis and immunotherapy according to prior study^24^. As a result, the link between TMB and the aforementioned signature was examined in the current investigation. Figure 9A,B show the mutant genes in the two groups. Additionally, the TMB in the high-risk group and that in the low-risk group were contrasted. The results of the present study revealed a favorable relationship between TMB and the risk score (P < 0.05; Fig. 9C). The OS was compared between the high-TMB and low-TMB groups after the samples were further divided into these two groups (P < 0.05; Fig. 9D). The OS was lower in the group with high TMB compared with the group with low TMB. Furthermore, the whole patient cohort was divided into four groups (‘H-TMB + high risk’, ‘H-TMB + low risk’, ‘L-TMB + high risk’ and ‘L-TMB + low risk’) and the OS was compared. The prognosis of patients in the four different groups was significantly different (P < 0.001; Fig. 9E).

### Cuproptosis-related lncRNA signature and m6A methylation

**Figure 10:**
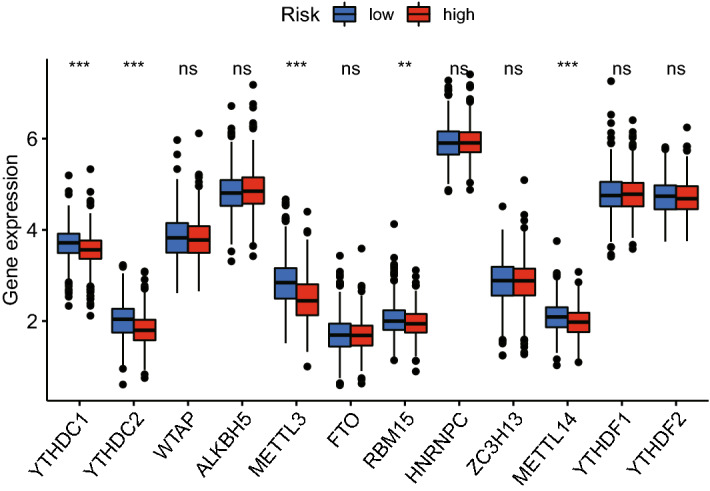
mRNA expression levels of 12 m6A methylation-related genes in the high- and low-risk groups.

Previous studies have reported that m6A methylation serves significant roles in immune system of various cancer types^25,26^ and that lncRNAs are associated with m6A methylation^27,28^. Therefore, 12 m6A methylation-related genes (METTL3, METTL14, WTAP, RBM15, ZC3H13, YTHDC1, YTHDC2, YTHDF1, YTHDF2, HNRNPC, FTO and ALKBH5) were selected and their mRNA expression levels in high- and low-risk patients were compared (Fig. 10). The findings indicated that the expression levels of YTHDC1, YTHDC2, METTL3, RBM15 and METTL14 of high- and low-risk patients differed considerably.

## Discussion

Lung cancer seriously threatens human lives worldwide due to its high morbidity and mortality rates^29^. However, traditional therapy has not provided patients in advanced stages or recurrence with the expected benefits^30^. According to the latest research, copper targets lipoylated TCA cycle proteins, causing lipoylated proteins to congregate and related iron-sulfur cluster proteins to disappear. This causes proteotoxic stress, which ultimately leads to cell death. Therefore, cuproptosis was used to describe this copper-dependent cell death. A variety of human tumor cells show a strong association with cuproptosis, indicating that copper ionophore intervention should concentrate on malignancies with this metabolic profile^5^.

Increasing evidence suggests that lncRNAs play a wide range of roles in fundamental and important biological processes, including cell differentiation, immune response, cell cycle control, imprinting and splicing^31,32^. And our study investigated the involvement of lncRNAs in lung cancer cuproptosis. Noteworthy, with the development of bioinformatics, computational model has become a common and effective model used in medical researches. Among a great number of computational models, non-coding (ncRNA) related models play key roles in complex human diseases, such as cancer, Alzheimer’s disease, Heart failure (HF), etc^33–35^. As a result of these, computational biology and ncRNA shows great potential ability in prediction of different human diseases.

NSCLC is a common classification to explore the mechanism of lung cancer (mainly LUAD and LUSC). Recently, there are several bioinformatics studies revealed LUAD had a relation with cuproptosis and TIME^36–38^. However, there is no research to explore the correlation between cuproptosis, TIME, and NSCLC. To predict the prognosis and examine the association with TIME and find the lncRNAs contribute to NSCLC, our study developed a novel and more precise cuproptosis-related lncRNA signature.

**Figure 11:**
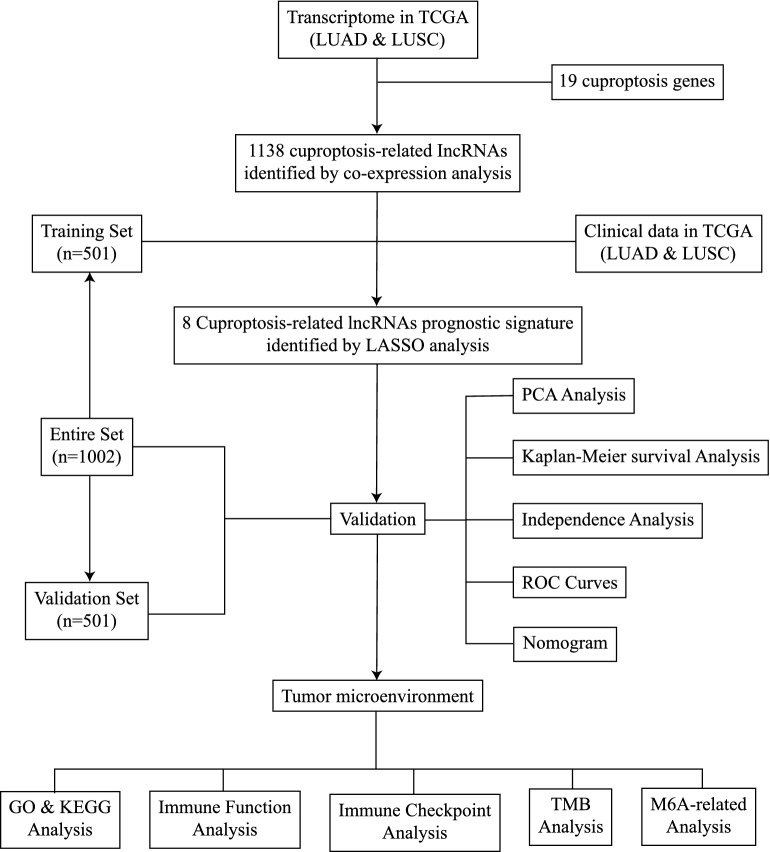
Flow diagram.

Using transcriptome information from the LUAD and LUSC cohorts in TCGA, a cuproptosis-related lncRNA signature was created in the present study (Fig. 11). A total of 19 genes were selected to be cuproptosis genes based on previous studies, and 16,773 lncRNAs were screened out from TCGA LUAD and LUSC transcriptome data. Subsequently, a co-expression test was performed, and finally 1138 lncRNAs were determined to be cuproptosis-related lncRNAs (coefficient > 0.4; P < 0.001). After analyzing 1138 lncRNAs related to cuproptosis using the Cox and LASSO methods, 8 lncRNAs (AL133445.2, LINC02635, AC026355.2, OGFRP1, AL161757.2, PTPRG-AS1, RPARP-AS1 and AC092127.2) were highlighted. The cuproptosis-related lncRNA signature was created using these 8 lncRNAs. Among these 8 lncRNAs, OGFRP1 has been reported to be the oncogene in NSCLC^39^. PTPRG-AS1 was reported to increased the proliferation in LUAD^40^ and reduced the radiosensitivity in NSCLC as well^41^. These previous studies confirmed that 8 lncRNAs have the potential to affect the development of LUAD and LUSC. Based on the signature, the risk score of every patient could be determined. According to the median of the risk scores, samples were divided into high- and low-risk groups. Additionally, the risk score and other clinical markers were used in univariate and multivariate analyses. The findings of the present study demonstrated that the risk score might be used as a standalone indicator to forecast the prognosis of patients with LUAD or LUSC. The ROC curves and PCA were used to assess the predictive power of the built signature, and a nomogram was created by combining the risk score with these clinicopathological characteristics (gender, age, stage, T stage, N stage). As the clinical data of M stage were not enough, M stage was not used to construct nomogram. Additionally, clinical subgroups (stage I-II and III-IV; age < 65 and ≥ 65 years; female and male; LUAD and LUSC) also demonstrated strong prediction for the signature.

In order to ascertain the mechanism underlying the present study's cuproptosis-related lncRNA signature's prediction, GO and KEGG enrichment analyses were performed using the differently expressed genes in the high- and low-risk groups. The enrichment analyses showed that the signature was associated with immune functions. Immunotherapy has currently been applied for the treatment of a variety of tumor types, including lung cancer^42^, melanoma^43^, cervical cancer^44^ and liver cancer^45^. Immune checkpoints, for instance programmed cell death protein 1 (PD-1) and cytotoxic T-lymphocyte-associated protein 4 (CTLA-4), have the ability to encourage anticancer immune function^46,47^. Subsequently, in present study, immunological checkpoints, immune functions and immune cell infiltration were compared in two groups (high- and low-risk). PD-1 and PD-L1 did not show significantly different expression between the two groups. However, the traditional checkpoint CTLA-4 and a number of other checkpoints were expressed differently in the two groups. This result may contribute to finding novel immune checkpoint inhibitors to treat lung cancer. Additionally, the relationships among TMB, risk score and prognosis in patients were identified. The findings of the present study demonstrated a positive link between the risk score and TMB and a negative association between TMB and the OS of patients with LUAD and LUSC. This may also explain how the survival rate of patients with LUAD and LUSC can be predicted using the cuproptosis-related lncRNA profile created in the present study.

In the majority of eukaryotic lncRNAs and mRNAs, m6A methylation is an essential RNA post-transcriptional modification^48^. Three different types of regulators, writers (methyltransferases), erasers (demethylases) and readers, can be used to influence the target RNA's stability, splicing, destruction, translation and processing (binding proteins)^49^. According to some research, numerous malignancies are associated with m6A methylation^50^. Additionally, multiple studies have indicated that m6A methylation is involved in the control of the immune response and TIME^51,52^. Therefore, the expression levels of m6A methylation-related genes were compared between the high- and low-risk groups. The results illustrated that YTHDC1, YTHDC2, METTL3, RBM15 and METTL14 were differently expressed in the two groups.

The accuracy of the signature was demonstrated in several sample sets in the current analysis, demonstrating the dependability of the signature. Furthermore, the link between the risk score and immune cell infiltration, immunological functions, immune checkpoints, TMB, and m6A methylation was found as another aspect of the signature's predictive process. However, the present study does have several drawbacks. To further evaluate the capacity of this signature to predict outcomes, more lung cancer patients, including LUAD, LUSC, large cell lung cancer, small cell large cancer, must take part in clinical trials. Furthermore, bioinformatics and non-coding RNA, such as miRNA, lncRNA, play key roles in different human diseases.

## Conclusion

In summary, in the present study, a cuproptosis-related lncRNA signature that could reliably predict prognosis and was associated with TIME in patients with LUAD and LUSC was developed. For patients with LUAD and LUSC, the built signature may offer a more thorough theoretical foundation and aid in exploring the more intricate cuproptosis mechanism.

## Supplementary Information

Supplementary Figure 1.

Supplementary Table S1.

Supplementary Table S2.

Supplementary Table S3.

Supplementary Table S4.

## Data Availability

The transcriptome and clinical data of LUAD and LUSC are available from the TCGA database 512 (https://portal.gdc.cancer.gov/).
