# Supplementary material for: Construction and validation of a cuproptosis-related lncRNA signature for the prediction of the prognosis of LUAD and LUSC
Source: Sci Rep. 2023 Feb 11;13:2477. doi: 10.1038/s41598-023-29719-1 (PMC9922262; doi:10.1038/s41598-023-29719-1)
Supplement: Supplementary file 1 — Supplementary Figure 1. [file 41598_2023_29719_MOESM1_ESM.docx]

**Supplementary Figure 1.** The univariate analysis result of 133 cuproptosis-related lncRNAs.


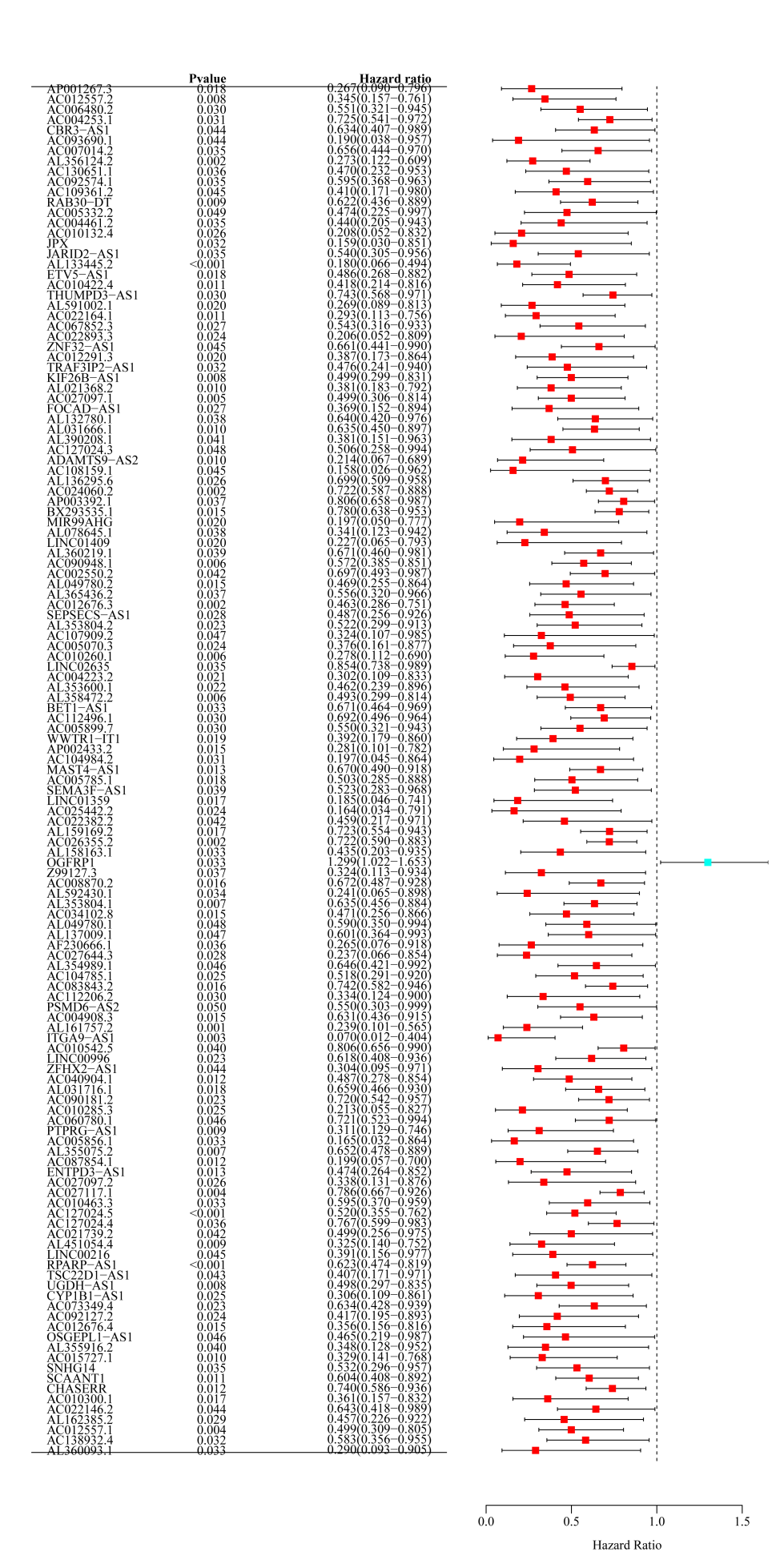


**Supplementary** **Table 1.** Correlation Test result of 1,138 lncRNAs and cuproptosis-related genes.

**Supplementary Table 2.** The univariate analysis result of 133 cuproptosis-related lncRNAs.

**Supplementary Table 3.** The details of 155 differentially expressed genes between high- and low-risk samples

**Supplementary Table 4.** The details of infiltrated immune cells.
